# Freeze-all, for whom, when, and how

**DOI:** 10.1080/03009734.2020.1746870

**Published:** 2020-04-14

**Authors:** Paula Celada, Ernesto Bosch

**Affiliations:** Instituto Valenciano de Infertilidad, Valencia, Spain

**Keywords:** Controlled ovarian stimulation (COS), cryopreservation, embryo implantation, freeze-all, fresh embryo transfer, frozen embryo transfer (FET), in vitro fertilization (IVF), segmentation

## Abstract

**Background:** The ‘freeze-all’ practice refers to the cryopreservation of all mature oocytes or viable embryos after ovarian stimulation. The development of the vitrification technique has been crucial to make this approach a reality, since it increases the post-thaw survival rates and permits comparable implantation rates with fresh embryos. Nonetheless, as implantation probabilities are comparable to fresh embryo transfer in normo-responder patients, the freeze- all strategy has demonstrated no benefits overall.

**Method:** Narrative review in which we give an overview of this approach, discuss recent advances in the field, as well as for whom, when and how it is recommended to emply the freeze-all technique.

**Results:** However, there is some clinical evidence that shows its feasibility. Thus, it has been demonstrated that elevation of progesterone at the end of ovarian stimulation decreases the implantation rates after the transfer of day 6 blastocysts in fresh and some uterine pathologies; freeze-all is also the preferred option for patients undergoing pre-implantation genetic testing, since there is an improvement of the results and it allows for inclusion of all blastocysts of the cohort. In high responders, the freeze-all strategy optimizes the response whilst also minimizing the risk of ovarian hyperstimulation syndrome.

**Conclusion:** Due to the different cases that a reproductive expert might encounter, it is essential to highlight benefits and drawbacks of this practice.

## Introduction

The success of embryonic implantation relies on a perfect synchrony between embryo status and endometrial receptivity. This process involves uterine, embryonic, and environmental factors that can contribute to a healthy uterine microenvironment (1). Improvements in assisted reproduction techniques (ART) allow for the development of new tools and technologies to preserve the safety and optimize treatment results.

The ‘freeze-all’ practice refers to the cryopreservation of all mature oocytes or viable embryos after ovarian stimulation (OS). Its application was primarily illustrated in protocols to avoid ovarian hyperstimulation syndrome (OHSS) by delaying implantation (2,3). The term ‘segmentation’ of *in vitro* fertilization (IVF) treatment was used by elective cryopreservation and postponed embryo transfer pioneers to outline the process where ovarian stimulation and oocyte/embryo retrieval are separated from the process of embryo transfer (2). Recently, elective frozen embryo transfer (eFET) emerged as an improved term that better describes the entire process (4).

In recent years, IVF segmentation, i.e. the ‘freeze-all’ strategy, has been proposed to increase cycle outcomes by avoiding the transfer of embryos in the same OS cycle, since the endometrium could be less receptive than in natural or artificial endometrial preparations. Several studies performed in recent years have suggested that transferring embryos in a later natural or artificial cycle to an endometrium that has not been exposed to high doses of exogenous gonadotropins could be a suitable approach (5–9). Indeed, the aforementioned reports have observed that the clinical pregnancy rate per transfer increases in the cryopreservation group. This strategy is available due to the improvement of cryopreservation technology in the last decade, which has reached the extent of 80–100% post-thaw survival rates and comparable implantation rates with fresh embryos (10–12).

## Freeze all for all strategy

Several studies have been published to support the ‘freeze-all’ procedures as a possible strategy in all IVF cycles (10). However, even though there might be a benefit from using this approach, the evidence available does not justify a change. A careful analysis of previous studies shows that these data have a number of limitations. In most cases, the patients included highlighted a trend to high ovarian response, which is associated with higher risk of OHSS, and higher probability of having high oestradiol and progesterone levels at the end of stimulation.

In the study by Shapiro et al. (7), 70 patients that had submitted to elective cryopreservation showed a significantly better ongoing pregnancy rate (OPR) than the 67 who underwent fresh embryo transfer. However, several limitations and biases have been put forward regarding the small number of patients, the high pregnancy rates in the cryopreservation cycles, and the presence of dual triggering (referred as the effect of co-interventions).

In a much larger study, Roque et al. (13) observed a significantly higher OPR in the ‘freeze-all’ cycles than in fresh transfers. However, the study groups were not comparable as patients that received the ‘freeze-all’ approach showed a significantly greater ovarian response and higher progesterone levels on the day of triggering (1.66 ± 0.14 versus 0.70 ± 0.27). This suggests that these patients were subject to this strategy to avoid the negative impact of high progesterone on endometrial receptivity. Therefore, the benefit of the elective cryopreservation has been verified in studies including high responders.

Roque et al. (13) also compared outcomes between fresh embryo transfer and freeze-all cycles in correlation to the number of retrieved oocytes. In the group with 4–9 retrieved oocytes there were no differences in OPR between the fresh and freeze-all group (31% and 33%, respectively). However, comparing the fresh and freeze-all groups, a better outcome was obtained in the freeze-all group when large numbers of oocytes were retrieved (34% and 47%, respectively). This may be taken to indicate that when high-response patients are excluded, there is no benefit of a freeze-all strategy.

These results are in agreement with a retrospective study performed in our centre comprising a cohort of 882 women aged 20–44 years undergoing their first or second IVF/ICSI cycle. Our study highlighted no benefit on live birth rates (LBR) of freeze-all versus fresh transfer in normo-ovulatory women undergoing IVF (14). We excluded patients with a risk of OHSS, high responders, and women with high progesterone levels on the day of trigger, since these subgroups have had improved outcomes with frozen embryo transfer (FET). There were no differences between FET and fresh embryo transfers in normal responders (4–20 oocytes) regarding implantation, clinical and ongoing pregnancies, and live births (14).

Similar results were highlighted recently in two large randomized controlled trials (RCT) (15,16). These studies showed that there is no benefit in LBR of the freeze-all strategy when comparing it with fresh embryo transfer in normo-ovulatory women undergoing IVF. More importantly, the Chinese study also displayed that the risk of moderate or severe OHSS was significantly decreased in the freeze-all group (15). In recent years, 11 randomized trials including 5379 patients who underwent IVF/ICSI were analysed in a meta-analysis (4). When the different subgroups were scrutinized, LBR were positively affected only in hyper-responders and in pre-implantation genetic testing (PGT-A) cycles. There was no difference in LBR in normo-responders.

## When to freeze-all

### PGT-A programmes

Recent developments in IVF practices such as extended embryo culture, vitrification, and trophectoderm biopsy, in combination with recently introduced technologies in PGT-A, have improved OPR as regards selective transfer of euploid blastocysts (17). Two transfer strategies for euploid embryos currently used in clinical environments are the employment of vitrified/warmed (‘freeze-all’) or fresh embryos for the first embryo transfer (ET). The freeze-all strategy involves cryopreservation of all embryos after biopsy, while performing a pre-implantation genetic screening (PGS) of the whole cohort (day 5 and day 6 embryos) in preparation for a frozen embryo transfer. The fresh strategy requires expanded blastocysts to be available on the morning of day 5, and culture overnight to await PGS results for a fresh embryo transfer of euploid embryos before noon on day 6. However, there is a negative impact of controlled ovarian stimulation on embryo–endometrium synchrony when transferring embryos on day 6 in a fresh autologous cycle. This is due to the impaired implantation of day 6 blastocysts in fresh transfers (8).

In this context, the freeze-all strategy seems to be more suitable as it allows time for PGT-A results to reach the clinician, and the transfer of a euploid embryo would be performed in a subsequent cycle. This hypothesis was confirmed in a RCT in which 179 patients underwent IVF treatment and PGT-A (18). OPR (80% versus 61%) and LBR (77% versus 59%) were significantly higher in the frozen group compared with the fresh group. Thus, freeze-all is the preferred option for patients undergoing PGT-A, since there is an improvement of the results and it allows inclusion of all blastocysts of the cohort.

### OHSS prevention

Ovarian hyperstimulation syndrome (OHSS) is a life-threatening complication of ovarian stimulation in IVF cycles. Elective cryopreservation of all embryos and their subsequent transfer in non-stimulated cycles may be employed to avoid the endogenous hCG increase in fresh transfer cycles, preventing the risk of OHSS. One RCT of 125 patients showed that after cryopreservation there was a lower incidence of OHSS than in controls with fresh embryo transfers (0 events versus 4 events, respectively) (19).

Another large RCT performed in polycystic ovarian syndrome (PCOS) patients (*n* = 1508) confirmed that the frequency of OHSS was significantly lower in freeze-all and subsequent FET cycles versus fresh transfer cycles (1.3% versus 7.1%) (20). Furthermore, LBR after the first embryo transfer was higher with this strategy when compared with fresh embryo transfer (49.3% versus 42.0%) in PCOS patients. Indeed, these studies support the recommendation of the American Society for Reproductive Medicine, which states that the freeze-all strategy in high responders optimizes the response whilst also minimizing the risk of OHSS (21).

### Elevated progesterone

During the last 30 years it has been discussed if the success of ART could be influenced by the increase in serum progesterone concentrations during ovarian stimulation. There is evidence to suggest that there is a decrease in implantation rates following fresh embryo transfer (22–24), but also studies reporting no association (25,26)

It has been repeatedly demonstrated that elevated progesterone negatively affects implantation by impairing endometrial receptivity (27). However, the association between elevated progesterone and embryo quality is still a matter of debate.

While it has been generally accepted that there is no negative impact on oocyte quality or OPR in recipients of donated oocytes (23,28), a recent retrospective study of 3400 cycles has postulated that there may be a detrimental effect (29). This study concluded that high serum progesterone concentrations at the end of the follicular phase are associated with a decrease in embryo utilization rates and cumulative live birth rates (CLBR) after both fresh embryo transfer and use of the freeze-all strategy. The negative influence that elevated progesterone may have on CLBR suggests that the freeze-all strategy is insufficient to solve the problem.

### Day 6 blastocysts

As mentioned above, there is an impaired implantation of day 6 blastocysts in fresh transfers. However, if day 6 blastocysts are transferred in a freeze–thaw cycle it is more likely to result in pregnancy with no differences of OPR between day 5 and day 6 blastocysts (8,30). The embryo–endometrium asynchrony might primarily be implicated in the impairment, with no effect on rapidly developing (day 5) blastocysts and a higher one in (day 6) blastocysts.

### Uterine pathology

There are different uterine pathologies that can be diagnosed during ovarian stimulation. They have all been associated with a decrease in fertility.

#### Polyps

Some studies suggest that polyps have a negative effect on fertility because they affect endometrial receptivity (31). If an endometrial polyp is identified during infertility evaluation, hysteroscopic polypectomy prior to treatment is recommended. Nonetheless, various strategies have been considered in the case of polyp detection during ART. These include embryo cryopreservation, hysteroscopy, and then transfer in a subsequent cycle (32).

#### Adenomyosis

A recent meta-analysis showed that adenomyosis seems to have a negative impact on ART outcome (33). Depot GnRHa for 3–6 months, administered alone or in combination with cytoreductive surgery, has been the most applied approach; however, there is poor evidence on the specific outcome in ART after such treatment. A recent study showed that vitrified embryos and transfer after treatment with a GnRH agonist tended to increase the pregnancy rates (34).

#### Hydrosalpinx

Some meta-analyses have demonstrated impaired pregnancy outcomes in patients with hydrosalpinx. Salpingectomy before embryo transfer improved their chance of achieving a birth after IVF treatment (35).

### Random-start ovarian stimulation

During the menstrual cycle, two waves of follicular growth may occur (36). New strategies for ovarian stimulation and, in particular, the random-start ovarian stimulation protocols have followed this new perspective on ovarian function. At present, there are few reports on the efficacy of random-start protocols. Preliminary results highlighted similar rates of total numbers of oocytes, as well as metaphase II oocytes obtained and fertilization rates in early follicular and random-start protocols (37). Furthermore, there was no difference in the probability of achieving an euploid blastocyst (38). Likewise, improvements of embryo and oocyte vitrification have permitted the development of new ideas such as total ‘disarticulation’ between ovarian stimulation and embryo transfer.

In cancer patients, random start is currently performed to minimize delays between ovarian stimulation and cancer therapies, with no difference in oocyte yield between conventional and random-start protocols (39). Another approach is the dual stimulation. This strategy can be useful in patients with poor ovarian response in order to save time by continuing ovarian stimulation after the first oocyte retrieval, thereby performing two stimulations in the same cycle (38,40). In the event of a ‘non-conventional start’ stimulation, all the oocytes/embryos need to be cryopreserved and transferred subsequently, due to the asynchrony between endometrial receptivity and embryo development.

## Optimizing preservation

### Methods of preservation

Slow freeze and rapid thaw techniques in cryopreservation made the first successful pregnancies from frozen embryos a reality. However, the formation of crystals due to the solidification of water is the main issue together with altered intracellular morphology. Indeed, these technical aspects led to low success rates, and the improvement of elements related to cryopreservation did not improve the freeze-all approach. A crucial step in the field was the development of the vitrification technique, which combines ultrarapid cooling in combination with cryoprotective agents to increase viscosity and decrease the freezing point of the environment. Vitrification was found to be effective regardless of the developmental stage of the embryo (12); nonetheless a study by Kuwayama et al. (41) described the Cryotop^®^, which was later accepted as an efficient approach. Vitrification is considered to be superior to slow freezing, and it is now an established protocol for ART (42).

### Endometrial preparation for FET

Adequate endometrial preparation is mandatory for the success of FET. It is still debated which is the best protocol to prepare the endometrium.

FET preparation methods can be divided into artificial and natural cycles. In artificial cycles, endometrial proliferation is achieved by oestrogen supplementation. In natural cycles, endogenous oestrogens secreted during the follicular menstrual cycle enhance the development of the endometrium. Natural cycles can be achieved with spontaneous ovulation or with ovulation induction.

Recent reviews and meta-analyses concluded that there is no difference in LBRs following different methods of endometrial preparation for FET (43,44).

It is worthy of note, however, that these data are derived predominantly from retrospective studies. In this scenario, the superiority of one protocol over another should be accepted only after performing prospective randomized studies.

#### Hormonal replacement treatment (HRT) or artificial cycle

To achieve a receptive endometrium, HRT aims to mimic the natural cycle preparing the endometrium in two stages. The first step employs oestrogens to mimic the follicular phase of a natural cycle. In the second step, progesterone is added to oestrogen to mimic the luteal phase. The initiation of orally administered exogenous oestrogen on day one of the cycle is performed to suppress follicle growth and spontaneous ovulation. Oestradiol supplementation also results in adequate endometrial preparation.

Oestrogens may be administered orally, vaginally, and parentally (transdermal route). A Cochrane systematic review concluded that the type of oestrogen supplementation and route of administration had no effect on the success rates of FETs (45). Different oestradiol supplementation schedules have been developed. The most commonly reported optimal doses vary between 4 and 12 mg/d (44). Quite in contrast, progesterone administration and dosage are less standardized. Some retrospective studies suggest that the route of administration of progesterone is without impact (45,46). However, a recent RCT showed that vaginal progesterone alone (200 mg every 12 h) was inferior to protocols containing intramuscular progesterone (47). One retrospective cohort study of 346 women who underwent HRT FET concluded that doubling the standard dosage of progesterone vaginal gel 90 mg (Crinone) significantly increased LBR (48).

Measuring serum progesterone concentrations has received increased interest in recent years. Yovich et al. (49) found an optimal mid-luteal progesterone range between 22 and 31 ng/mL (70 and 99 nmol/L). Concentrations of progesterone below 22 ng/mL and above 31 ng/mL were associated with decreased implantation rates.

A recent prospective study in our centre confirmed that low serum progesterone concentrations on the day of transfer were associated with lower OPR (50). Interestingly, there was a wide range of progesterone concentrations, even though all of these patients received the same regimen of progesterone (400 mg/12 h from 5 days before embryo transfer). The results revealed a decrease of 20% in OPR in women with serum progesterone concentrations lower than 9.2 ng/mL (29 nmol/L) on the day of embryo transfer. Recently, Alsbjerg et al. (51) obtained results comparable with the study from Labarta et al. (50) described above. The Alsbjerg group found a decrease of 14% in OPR when progesterone was below 35 nmol/L (11 ng/mL) (Table 1).

**Table 1.** Comparison of optimal serum progesterone concentrations (nmol/L) for cryopreserved embryo transfers in artificial cycles in different studies.

Study	Patients included	Luteal phase support	P4 measurement	Optimal P4 values	Pregnancy outcomes under and over the cut-off
Yovich et al. 2015 (49)	529	Vaginal micronized progesterone (400 mg/8 h)	2–3 days after embryo transfer	>50 nmol/L; best range: 70–99 nmol/L	CPR: 44% (<50 nmol/L) versus 64% (70–99 nmol/L) (*P* = 0.005)
Labarta et al. 2017 (50)	244	Vaginal micronized progesterone (400 mg/12 h)	Day of embryo transfer	>29 nmol/L	OPR: 32.7% versus 52.8% (*P* = 0.016)
Alsbjerg et al. 2018 (55)	244	Vaginal micronized progesterone (90 mg/8 h)	9–11 days after embryo transfer	>35 nmol/L	OPR: 44% versus 58% (*P* = 0.02)

CPR: clinical pregnancy rates; OPR: ongoing pregnancy rates; P4: progesterone.

In HRT FET cycles there is no corpus luteum and, hence, no endogenous progesterone production. If a pregnancy occurs, oestrogen and progesterone must be continued until placental autonomy is established to replace the absent corpus luteum.

#### Natural cycle

Exposure to oestrogen and progesterone is a consequence of spontaneous follicle development and ovulation. This method is available only for patients with an ovulatory cycle. To assess embryo–endometrial synchronization it is essential to monitor the cycle with several pelvic ultrasound scans to confirm follicular development and ovulation timing. In a natural cycle, ovulation can occur physiologically by the spontaneous onset of a LH surge (natural cycle) or programmed by triggering ovulation exogenously by an injection of hCG (modified natural cycle).

The necessity for luteal phase support is still under debate. For natural cycles a RCT demonstrated a significantly higher LBR in the group receiving vaginal progesterone (400 mg twice a day from the day of embryo transfer) compared with those who received no progesterone support (52). For modified natural cycles, the results of several studies are conflicting, and there is considerable heterogeneity. Both prospective (53) and retrospective (54) studies failed to show any difference in terms of pregnancy outcome with or without progesterone support. However, another retrospective study suggested that luteal phase progesterone supplementation decreases the miscarriage rate and improves LBR (55).

## Safety of cryopreservation of embryos

Analyses of reproductive cryopreservation outcomes such as effects on pregnancies and on neonates highlight several consistent findings.

### Perinatal outcomes

#### Large for gestational age (LGA) and high birth weight

Some studies and meta-analyses have reported LGA after FET even when considering maternal age and birth order (56–58). Large epidemiological studies reported an increased risk of higher birth weight (birth weight >4000 g) and very high birth weight (birth weight >4500 g) in babies born after FET when compared with those born after fresh embryo transfer (59–61). The meta-analysis of Maheshwari et al. (62) confirmed these results for high birth weight (RR 1.85; 95% CI 1.46–2.33) and very high birth weight (RR 1.86; 95% CI 1.58–2.19).

Whether the higher risk of LGA is related to the freezing/thawing procedure *per se* or if other factors are involved remains unknown. Maternal BMI and parity were found to have a significant effect on the birth weight (63). Pinborg et al. (56) explored the risk of babies with LGA in a FET/fresh sibling cohort. The study suggested that children born after FET are at increased risk of LGA, even in the same mother. Therefore, this cannot be explained considering intrinsic maternal factors only. Aspects associated with the freezing/thawing procedures need to be considered as well.

To what extent there is an association between long-term *in vitro* culture and LGA remains unclear. Nevertheless, Mäkinen et al. (63) described such an association when analysing the birth weight of the children and the length of embryo culture. However, Wikland et al. (64) found no increased prevalence of LGA after transfer of vitrified blastocysts compared with slow-freeze cleavage stage transfer. The duration of the cryostorage of the vitrified blastocysts does not appear to affect pregnancy and neonatal outcomes (65). Nonetheless, the physiological mechanisms associated with the increased birth weight observed after FET need to be elucidated more in detail.

Recent evidence pointed out a possible epigenetic regulation of the cryopreservation process itself. As an example, genome-wide analysis has revealed differentially expressed miRNAs in FET placentae compared with placentae from fresh embryo transfers potentially involved in birth weight increase and perinatal complications (66).

#### Small-for-gestational age and low birth weight

It has been shown that there is a lower risk to be small for gestational age for babies born after FET compared to those born after fresh embryo transfer (RR 0.61; 95% CI 0.56–0.67) (62). More than 20 studies showed that there is also a decrease of the probability of low birth weight (less than 2500 g) in babies born after FET (62).

#### Preterm delivery (delivery at less than 37 weeks)

There are several studies showing that babies born after FET possess a lower risk of prematurity (58–61). The recent meta-analysis of Maheshwari et al. (62) confirmed these results, showing a reduction on relative risk of prematurity (RR 0.90; 95% CI 0.84–0.97).

#### Others

As regards some neonatal outcomes such as antepartum haemorrhage, admission to the neonatal intensive care unit, congenital abnormalities, and perinatal mortalities, there are no differences reported between frozen and fresh transfer strategies (62).

### Obstetric outcomes

Studies from Sweden (57) and Japan (60) highlighted the increased risk of pregnancy-induced hypertension and pre-eclampsia in singleton pregnancies following frozen–thawed cycles compared with fresh cycles and spontaneously conceived pregnancies. A large study in a Nordic population revealed a consistently higher risk of hypertensive disorders in pregnancies after FET even when compared with fresh cycle pregnancies in the same mother (67). This may be taken to indicate that the association cannot be attributed only to maternal factors. However, when FET was performed in the natural cycle there were no differences in pre-eclampsia or hypertensive disorders between FET and fresh embryo transfer (15). This suggests that the endometrial preparation protocol might have an impact on the obstetric outcomes (4).

## Cost-effectiveness of FET

Even though the quality of frozen embryos and the implantation probabilities are comparable to fresh embryo transfer (12,68,69), the overall cost of fertility treatment must be considered since fresh transfer is immediate. There is a need to consider the absence of direct costs of cryopreservation, additional medication, and subsequent FET cycles (18).

Roque et al. (70) have shown that the freeze-all approach has a lower cost than fresh transfer by analysing two scenarios in a cost-effectiveness analysis. Another study by Papaleo et al. (71) observed no cost difference between the freeze-all approach and fresh blastocyst transfer per live birth. The authors concluded that the cost similarity is due to insignificant additional expenses such as vitrification, endometrial priming, and monitoring versus fewer embryo transfers needed to obtain pregnancy.

When studying normal responder patients, a decision tree mathematical model showed that a single freeze-all cycle possessed an increased cost effectiveness when compared with a single fresh cycle even with the addition of a secondary supernumerary FET (72). On the other hand, in non-PCOS women undergoing IVF/ICSI, it was highlighted that there is a similar average cost per couple between the freeze-all approach and the fresh embryo transfer. From a patient perspective, other factors might be crucial to decide on a freeze only or fresh embryo transfer (73).

## Conclusions

**Figure 1.**
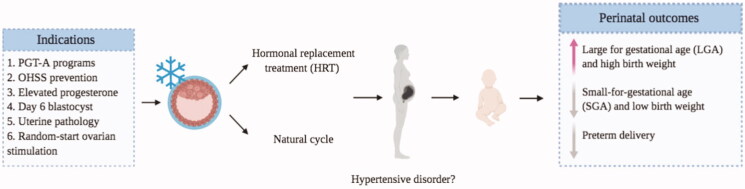
Embryo cryopreservation overview.

The availability of a freeze-only strategy in the armamentarium of IVF provides a number of opportunities to optimize the overall outcome in several every-day situations in daily practice. While the indiscriminate use with the goal to improve LBR in unselected populations cannot be proven, the appropriate indication of this strategy makes it possible to overcome a handful of obstacles that could lead to suboptimal results in terms of efficacy and safety. It is crucial that every practitioner knows and understands these indications, in order to provide the best possible health care (Figure 1).

Whether future findings will change the current picture is unknown. It cannot be discounted that new insights particularly related to obstetric and perinatal outcome may lead to a decrease in the use of freeze-only, or to moving towards the natural cycle for endometrial preparation.
